# Supplementary material for: Atomic model for core modifying region of human fatty acid synthase in complex with Denifanstat
Source: Nat Commun. 2023 Jun 12;14:3460. doi: 10.1038/s41467-023-39266-y (PMC10258763; doi:10.1038/s41467-023-39266-y)
Supplement: Supplementary file 1 — Supplementary Information [file 41467_2023_39266_MOESM1_ESM.pdf]

Supplementary Information

Atomic model for core modifying region of human fatty acid synthase in complex with Denifanstat

S. M. Naimul Hasan[1], Jennifer W. Lou[1], Alexander F.A. Keszei[2], David L. Dai[1], Mohammad T. Mazhab-Jafari[1,2]

[1]Department of Medical Biophysics, University of Toronto,
[2]Princess Margaret Cancer Center, University Health Network, Toronto, Ontario, Canada.
* Correspondence: mohammad.mazhabjafari@utoronto.ca

Supplementary Tables – Pages 2-3

Supplementary Figures – Pages 3-13

34 ## Supplementary Tables:

35 **Supplementary Table 1) Data processing and refinement statistics.** C2 refinement parameters are highlighted in parenthesis.

**Data Collection and Processing**	**FASN + NADPH**	**FASN + NADPH + TVB2640**
Microscope	Titan Krios G3	Titan Krios G3
Camera	FEI Falcon 4i	FEI Falcon 4i
Voltage	300 kV	300 kV
Magnification	75,000×	75,000×
Pixel size	1.03 Å	1.03 Å
Exposure	50.76 electrons/Å^2	50.76 electrons/Å^2
Number of Movies	4,016	4,801
Number of Frames per Movie	30	30
Defocus Range	0.6-2.5μm	0.6-2.5μm
Symmetry Applied	C1	C1 (C2)
Initial particle images (no.)	2,575,851	3,004,526
Final particle images (no.)	355,649	311,983
Map resolution (FSC = 0.5)	3.01 Å	2.94 Å (2.75 Å)
Map resolution (FSC = 0.143)	2.66 Å	2.64 Å (2.45 Å)
B-Factor applied	-50.0 Å^2	-50.0 Å^2 (-120 Å^2)
**Model Building**		
Modeling Software	Coot, Phenix	Coot, Phenix
Nonhydrogen atoms	16,253	15,387 (14,616)
Number of Residues build	2,172	2,036 (1,932)
Number of Ligand build	4	6 (6)
RMS (Bond)	0.003	0.004 (0.005)
RMS (Angles)	0.568	0.586 (0.641)
Ramachandaran Favored	98.05%	97.86% (96.98%)
Ramachandaran Allowed	1.95%	2.14% (3.02%)
Ramachandaran Outliers	0.00%	0.00% (0.00%)
C-beta outliers	0.00%	0.00% (0.00%)
Rotamer Outliers	1.99%	1.25% (0.00%)
All Atom Clashscore	10.19	10.78 (12.11)
MolProbity Score	1.76	1.66 (1.77)
Model-Map CC_mask	0.81	0.82 (0.82)
PDB ID	8EYI	8EYK (8GKC)

37

Supplementary Table 2) Deviation of backbone atoms between *h*FASN and *S. scrofa* FASN (PDB 2vz9). Rmsd was calculated using PyMOL align command for backbone atoms.

*h*FASN	RMSD (Å)
Overall	1.00
DH	0.58
ΨME	1.1
ΨKR	0.83
ER	0.65
KR	0.58

Supplementary Figure:

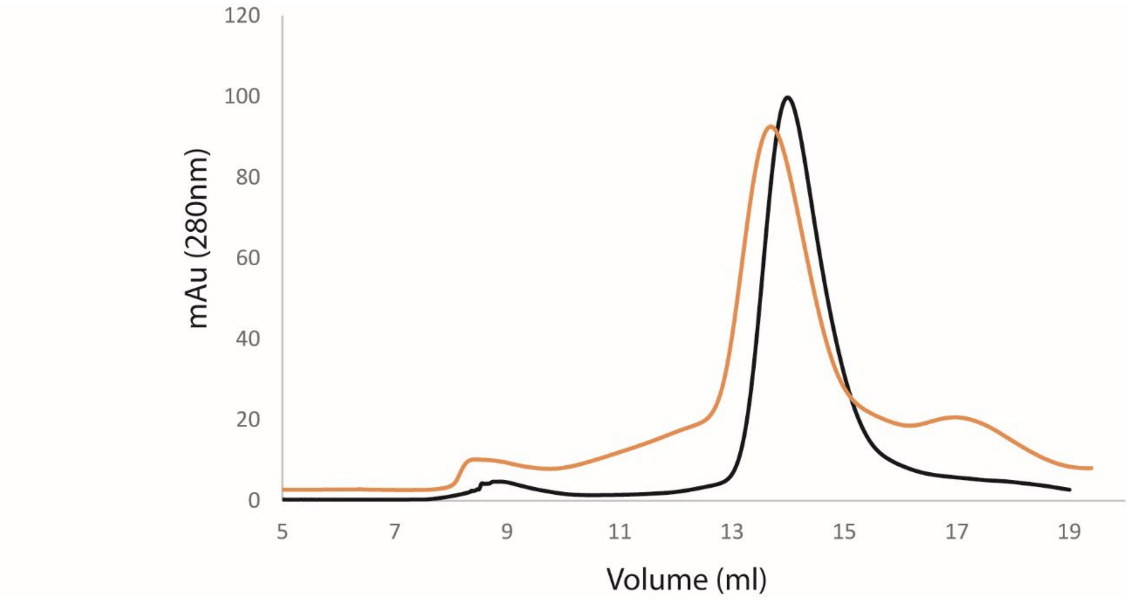

Supplementary Figure 1. Effect of insertion of TEV cleavage sequence between the condensing and modifying regions of *h*FASN on its elution profile. Size exclusion profiles of WT (black) and TEV inserted (orange) *h*FASN from Superose 6 increase 10/300 column.

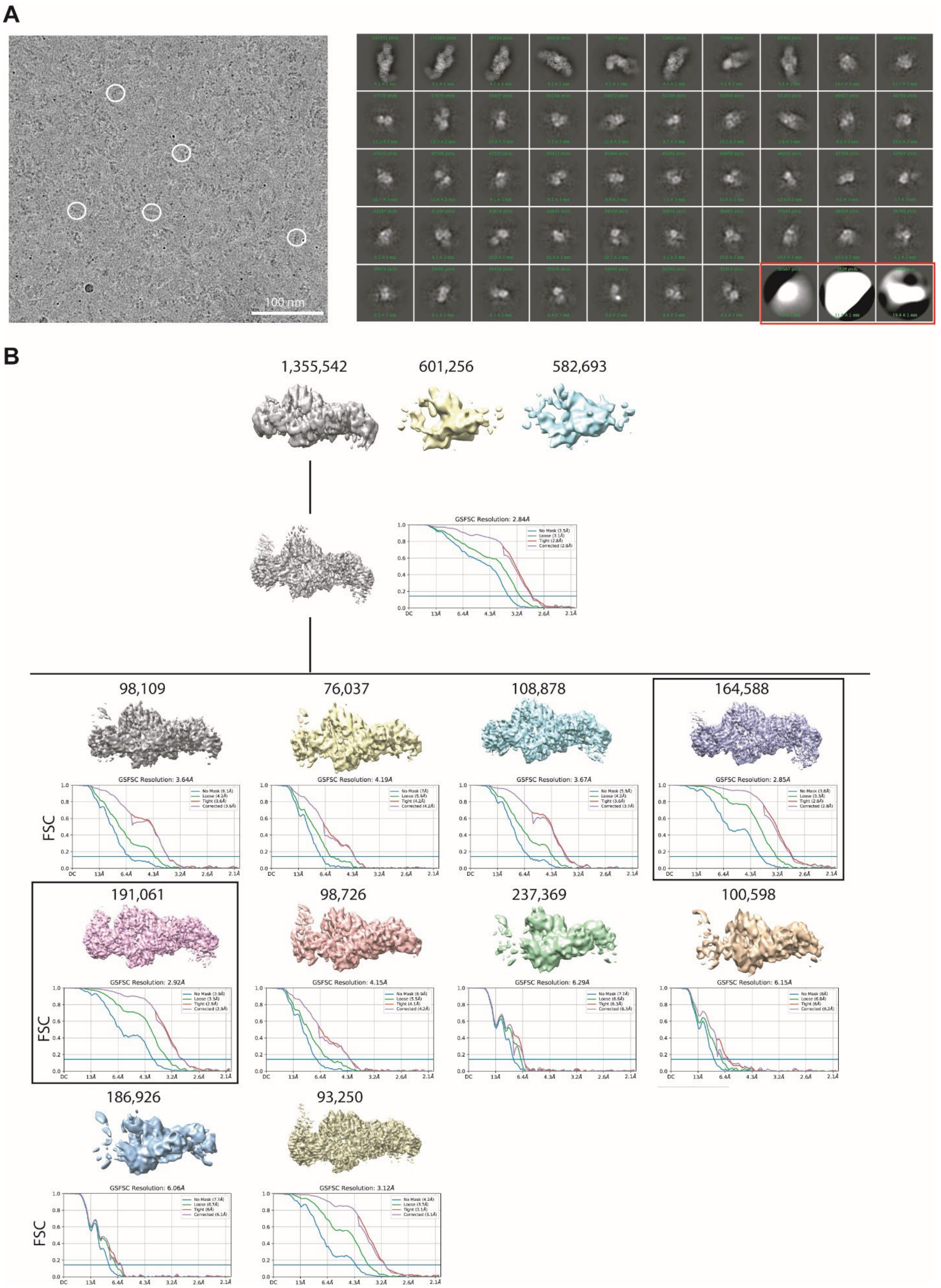
A
100 nm
B
1,355,542
601,256
582,693
GSFSC Resolution: 2.84Å
98,109
GSFSC Resolution: 3.64Å
76,037
GSFSC Resolution: 4.19Å
108,878
GSFSC Resolution: 3.67Å
164,588
GSFSC Resolution: 2.85Å
191,061
GSFSC Resolution: 2.92Å
98,726
GSFSC Resolution: 4.15Å
237,369
GSFSC Resolution: 6.29Å
100,598
GSFSC Resolution: 6.15Å
186,926
GSFSC Resolution: 6.06Å
93,250
GSFSC Resolution: 3.12Å
FSC

Supplementary Figure 2. Single particle images analysis of the modifying region of *h*FASN in complex with NADPH. A) A representative electron micrograph (from 4,016 micrographs) is shown with protein particles highlighted in white circles. Reference free 2D classifications demonstrated presence of multiple views for the modifying region of *h*FASN. Classes excluded are highlighted in red. **B)** 3D classification and reconstruction are shown. From the top: Selected 3D classes from heterogenous refinement are chosen for high-resolution homogenous refinement. Particles corresponding to this consensus refinement are then 3D classified using 10 classes without orientation search to identify the highest quality 3D map (*i.e.*, best global resolution and diversity of viewing directions, highlighted with rectangles). Particle number is shown for each 3D class.

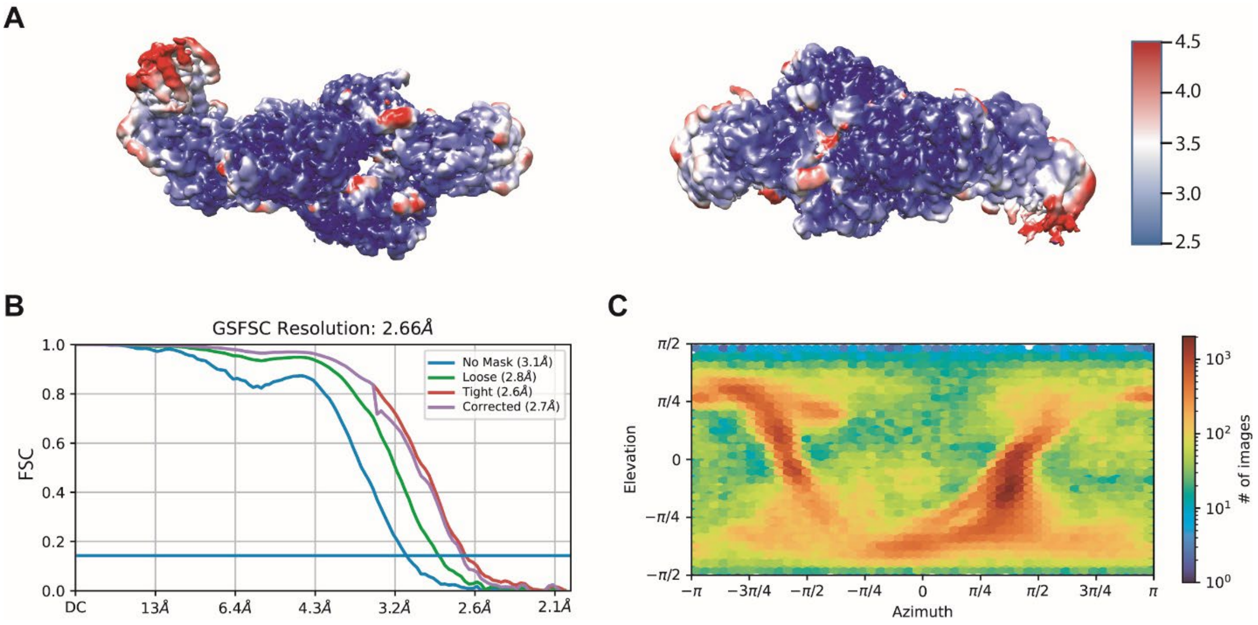

Supplementary Figure 3. High resolution 3D reconstruction of *h*FASN core modifying region in complex with NADPH. A) 3D cryoEM map colored based on local resolution estimate. Two orthogonal views are shown. **B)** Fourier shell correlation (FSC) curves using different masking schemes as well as mask corrected FSC (magenta) is shown to estimate the global resolution. **C)** Orientation distributions for particle data sets contributing the refined 3D reconstruction.

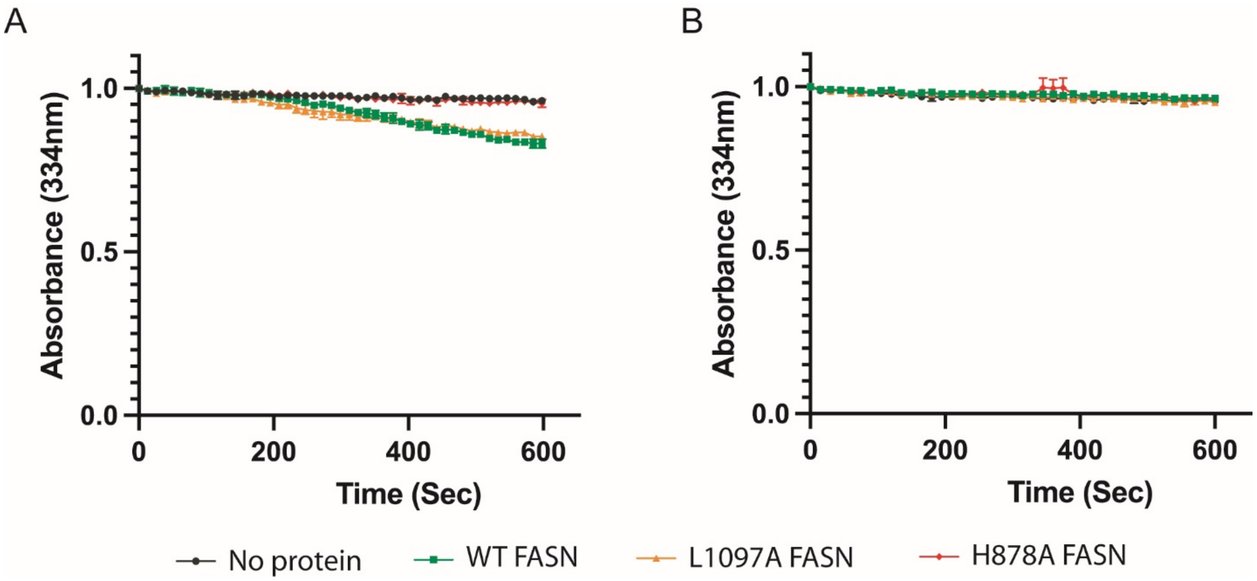

Supplementary Figure 4. DH activity assay using 3-hydroxybutyryl-CoA and NADPH. Assay is performed on **A)** full length TEV engineered *h*FASN and **B)** isolated modifying reigon of *h*FASN. The modifying region was expressed and purifed as an independent construct. Datapoints are average of two technical replicates.

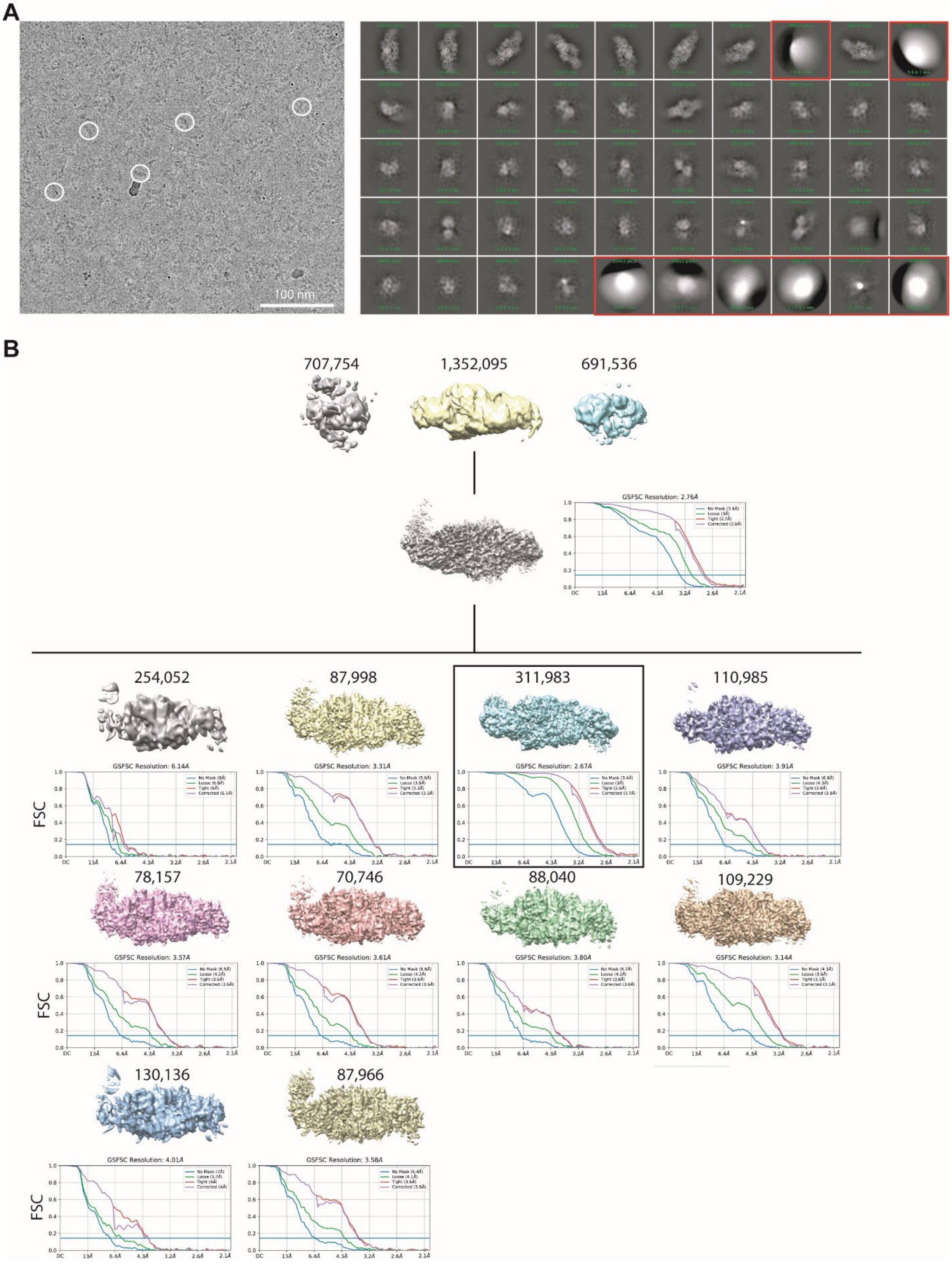
A
100 nm
B
707,754
1,352,095
691,536
GSFSC Resolution: 2.76Å
254,052
GSFSC Resolution: 6.14Å
87,998
GSFSC Resolution: 3.31Å
311,983
GSFSC Resolution: 2.67Å
110,985
GSFSC Resolution: 3.91Å
78,157
GSFSC Resolution: 3.57Å
70,746
GSFSC Resolution: 3.61Å
88,040
GSFSC Resolution: 3.80Å
109,229
GSFSC Resolution: 3.14Å
130,136
GSFSC Resolution: 4.01Å
87,966
GSFSC Resolution: 3.58Å
FSC
DC
13Å
6.4Å
4.3Å
3.2Å
2.6Å
2.1Å

Supplementary Figure 5. Single particle images analysis of the modifying region of *h*FASN in complex with NADPH and Denifanstat. Both panels **A)** and **B)** are as described in supplementary figure 2, except that template-based picked particles were first used in *ab initio* 3D reconstruction using three classes (panel B, top 3D volumes). A representative micrographs from 4,801 micrographs collected is shown in panel A. One *ab initio* class corresponding to *h*FASN was selected for high-resolution refinement followed by 3D classification without orientation search as in supplementary figure 2.

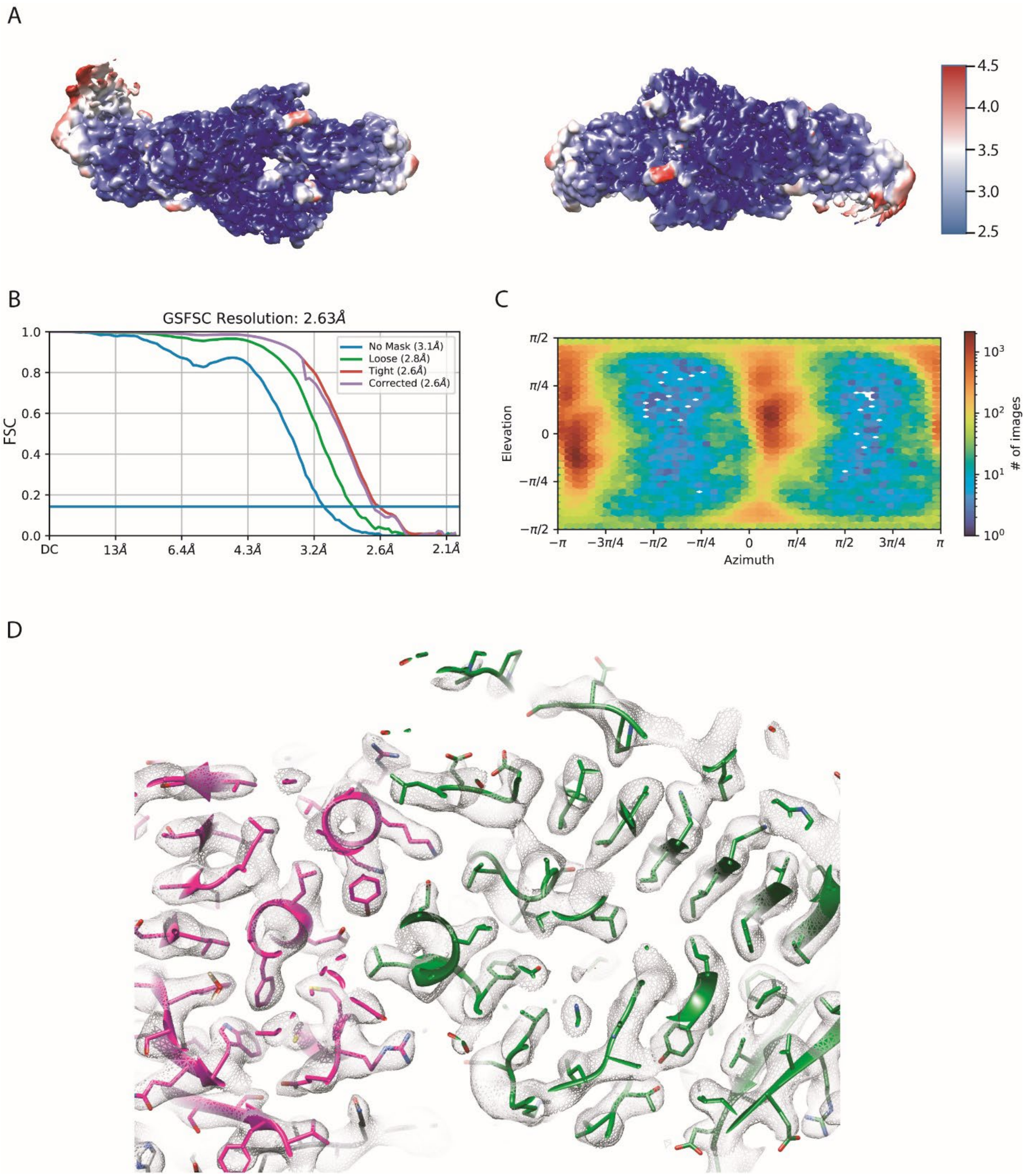

Supplementary Figure 6. High resolution 3D reconstruction of *h*FASN core modifying region in complex with NADPH and Denifanstat. Panels **A)**, **B)**, and **C)** are as described in supplementary figure 3. **D)** A slice through the model fitted into the cryoEM density map of the complex.

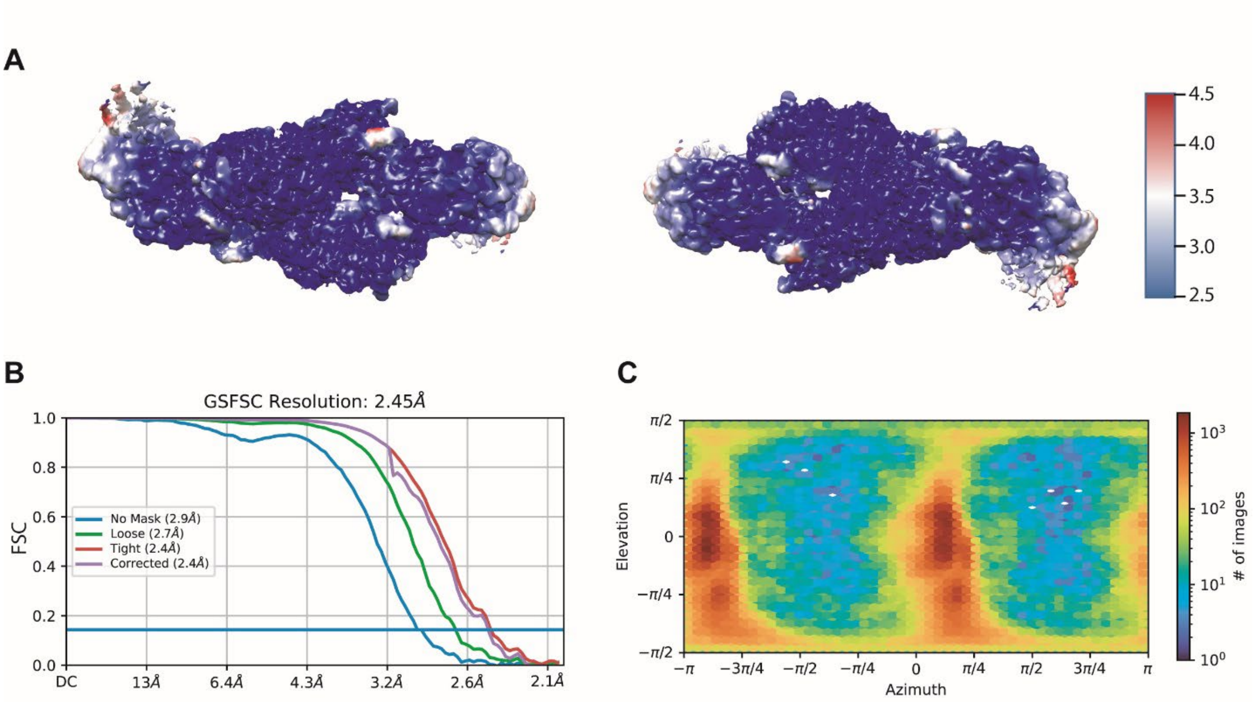

Supplementary Figure 7. 3D reconstruction of *h*FASN core modifying region in complex with NADPH and Denifanstat with C2 symmetry imposed. Panels **A)**, **B)**, and **C)** are as described in supplementary figure 5. Same particle image dataset was used for both C1 and C2 reconstructions.

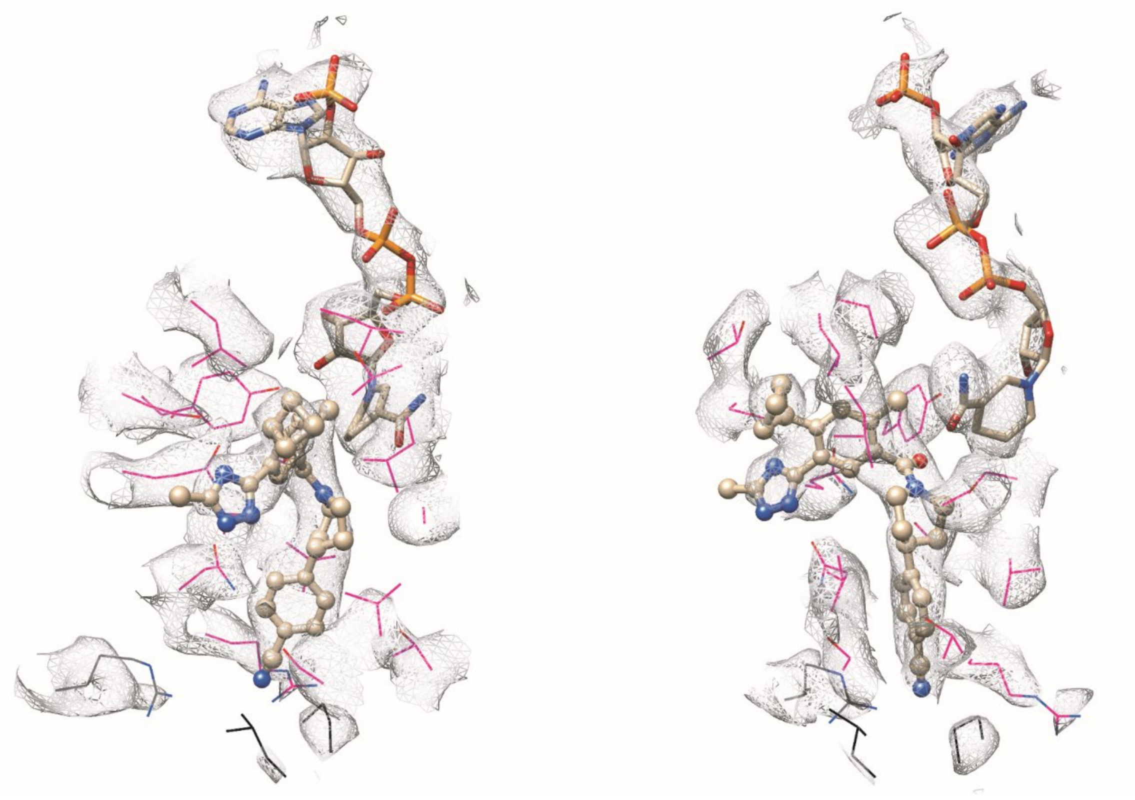

Supplementary Figure 8. cryoEM density of the drug binding site. Uncarved densities are shown around NADPH (stick representation) and Denifanstat (stick and ball representation). Density is shown from a cryoEM map with C2 refinement imposed. Protein residues proximal to Denifanstat are shown as lines. Two orthogonal views are shown.

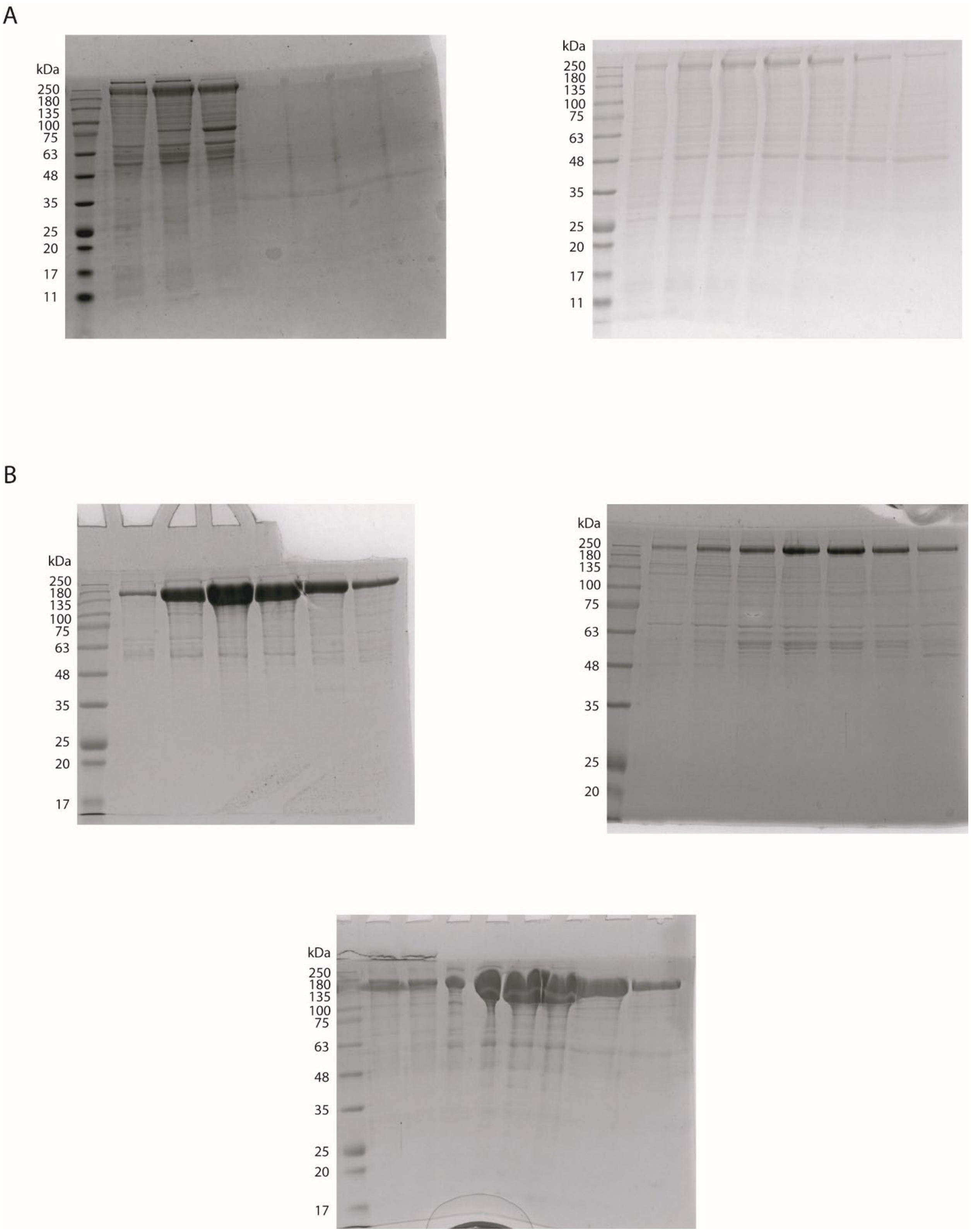

Supplementary Figure 9. Protein quality and purity assessments. A) Full length TEV inserted FASN constructs purified from two independent HEK293F cultures (i.e., transfected separately), shown in the left and right panels, respectively. Fractions corresponds to elution volumes from size

exclusion column. **B)** SDS-PAGE gels are shown for isolated modifying region of FASN expressed independently (top left, also shown in Fig 2D), full length FASN L1097A mutant with TEV insertion between the condensing and modifying region (top right), and isolated modifying region of FASN L1097A mutant expressed independently (bottom center). Fractions correspond to elution from size exclusion column. For all enzymatic assays, the elution fractions were pooled and used in the NADPH based oxidation assay. Each gel is a representative of one experiment.

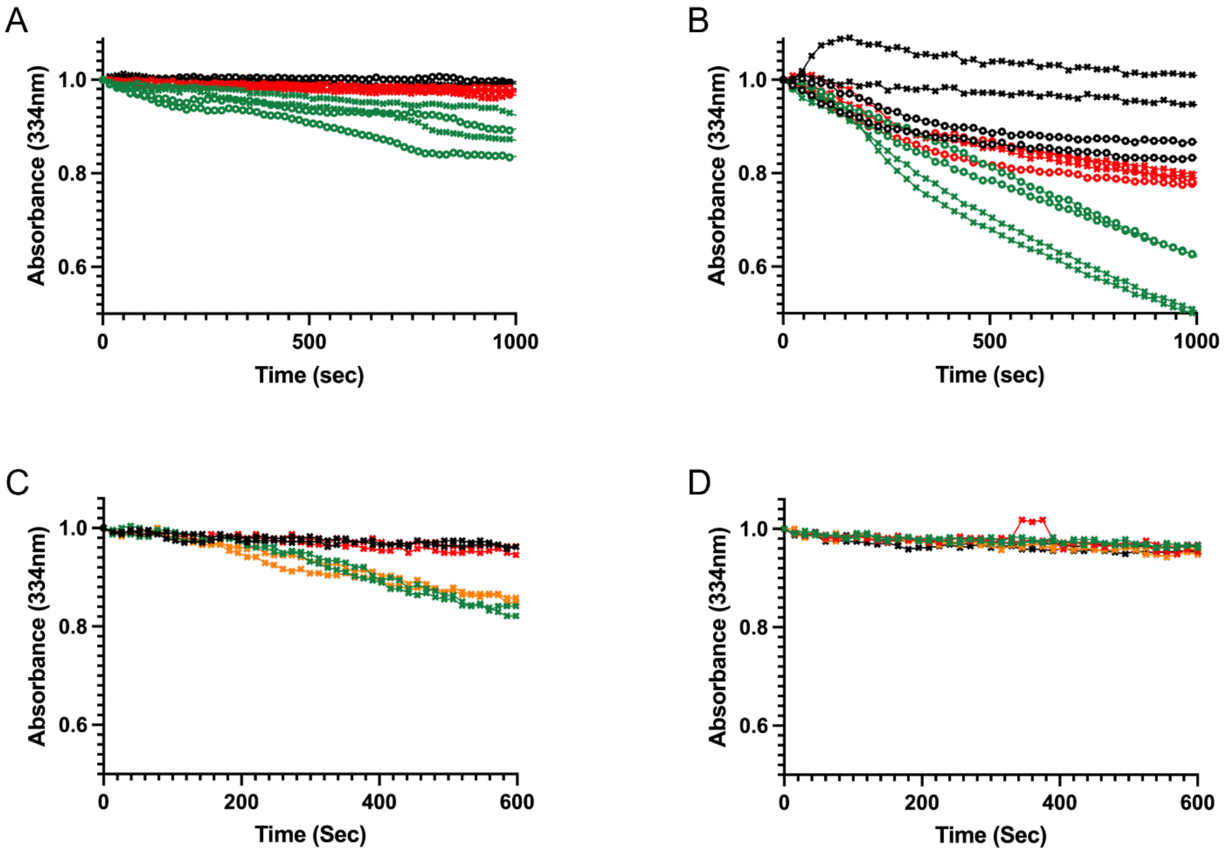

Supplementary Figure 10. FASN activity assay for each replicate. NADPH consumption is shown for each biological (coded as o) and technical (coded as ×) replicates reported in this study. Color coding is the same as figure 5A and supplementary figure 4. KR specific activity assay using trans-1-decalone and NADPH on **A)** full-length TEV engineered *h*FASN and **B)** modifying region expressed and purified as a truncated construct. DH activity assay using 3-hydroxybutyryl-CoA and NADPH on **C)** full-length TEV engineered *h*FASN and **D)** modifying region expressed and purified as a truncated construct. Source data are provided as a Source Data file.
