# Supplementary material for: Atomic model for core modifying region of human fatty acid synthase in complex with Denifanstat
Source: Nat Commun. 2023 Jun 12;14:3460. doi: 10.1038/s41467-023-39266-y (PMC10258763; doi:10.1038/s41467-023-39266-y)
Supplement: Supplementary file 3 — Description of Additional Supplementary files [file 41467_2023_39266_MOESM3_ESM.pdf]

Description of Additional Supplementary Files

File name: Supplementary Movie 1
Description: Bending motion hFASN core modifying region. Linear interpolation of the protein model describing the first principal component of 3DVA. Two orthogonal views are shown. Scale bar is 50Å.

File name: Supplementary Movie 2
Description: cryoEM density changes corresponding to the first principal component of the 3DV analysis. Residues corresponding to the loop at the entrance of the KR catalytic cavity is highlighted with ball and stick.

File name: Supplementary Movie 3
Description: Twisting motion hFASN core modifying region. Linear interpolation of the protein model describing the second principal component of 3DVA. Two orthogonal views are shown.

File name: Supplementary Movie 4
Description: cryoEM density changes corresponding to the second principal component of the 3DV analysis. Residues corresponding to the loop at the entrance of the KR catalytic cavity is highlighted with ball and stick.

File name: Supplementary Movie 5
Description: cryoEM density changes corresponding to the third principal component of the 3DV analysis. Residues corresponding to the loop at the entrance of the KR catalytic cavity is highlighted with ball and stick.
